# Redesigning an Electrochemical MIP Sensor for PFOS: Practicalities and Pitfalls

**DOI:** 10.3390/s19204433

**Published:** 2019-10-13

**Authors:** Giulia Moro, Davide Cristofori, Fabio Bottari, Elti Cattaruzza, Karolien De Wael, Ligia Maria Moretto

**Affiliations:** 1 Department of Molecular Sciences and Nanosystems, Ca’ Foscari University of Venice, Via Torino 155, 30172 Mestre, Italy; giulia.moro@unive.it (G.M.); dcristofori@unive.it (D.C.); cattaruz@unive.it (E.C.); 2 AXES Research Group, Department of Chemistry, University of Antwerp, Groenenborgerlaan 171, 2020 Antwerp, Belgium; fabio.bottari@uantwerpen.be (F.B.); karolien.dewael@uantwerpen.be (K.D.W.); 3 Centre for Electron Microscopy “Giovanni Stevanato”, Università Ca’ Foscari di Venezia, Via Torino 155, 30172 Mestre, Italy

**Keywords:** PFOS, molecularly imprinted polymer, biomimetic sensor, gold screen-printed electrodes, ortho-phenylenediamine

## Abstract

There is a growing interest in the technological transfer of highly performing electrochemical sensors within portable analytical devices for the in situ monitoring of environmental contaminants, such as perfluorooctanesulfonic acid (PFOS). In the redesign of biomimetic sensors, many parameters should be taken into account from the working conditions to the electrode surface roughness. A complete characterization of the surface modifiers can help to avoid time-consuming optimizations and better interpret the sensor responses. In the present study, a molecularly imprinted polymer electrochemical sensor (MIP) for PFOS optimized on gold disk electrodes was redesigned on commercial gold screen-printed electrodes. However, its performance investigated by differential pulse voltammetry was found to be poor. Before proceeding with further optimization, a morphological study of the bare and modified electrode surfaces was carried out by scanning electron microscopy–energy-dispersive X-ray spectrometry (SEM–EDS), atomic force microscopy (AFM) and profilometry revealing an heterogeneous distribution of the polymer strongly influenced by the electrode roughness. The high content of fluorine of the target-template molecule allowed to map the distribution of the molecularly imprinted polymer before the template removal and to define a characterization protocol. This case study shows the importance of a multi-analytical characterization approach and identify significant parameters to be considered in similar redesigning studies.

## 1. Introduction

In recent years, considerable efforts have been invested in the development of biomimetic electrochemical sensors for the indirect detection of non-electroactive targets, i.e., organic environmental contaminants. Among the so-called biomimetic modifiers, molecularly imprinted polymers (MIP) played a major role, providing a good sensitivity and selectivity and avoiding the drawbacks of other indirect detection strategies [1]. MIP target-mimetic cavities enable the pre-concentration of the target on the electrode surface, reaching limits of detection (LOD) in the nanomolar or picomolar range, and helping discriminate between large classes of structurally similar compounds, improving the selectivity [2,3,4]. Moreover, these modifiers can be directly integrated with the transducer by electropolymerization using electroactive functional monomers [5,6,7].

In spite of the large number of publications and the outstanding results reported in literature [8,9], the majority of these sensors do not reach the market and remain mere proof-of-concept studies. For the real commercial application of biomimetic sensors, the open challenges concern mainly: the miniaturization of the devices, the automation of sensor production, the development of user-friendly protocols and their applicability and reproducibility in real sample analysis. Therefore, there is a rising interest in understanding if modification protocols optimized on bulk electrodes can be easily redesigned on portable screen-printed electrodes (SPE).

This is the case for the biomimetic sensor for perfluorooctanesulfonic acid (PFOS, see [10,11] for an overview) developed on gold disk electrodes [12] that showed excellent performance (with a LOD lower than the lifetime health advisory limits of 0.04 nM [13]) and was successfully applied in water sample analysis. In this sensor, ferrocene carboxylic acid (FcCOOH) is used as a probe because of the non-electroactive nature of PFOS. Once the target is entrapped in the cavities of the non-conductive molecularly imprinted receptor, a decrease in the redox probe signal is recorded and related to the concentration of PFOS (inversely proportional). To assure the correct functioning of this indirect detection-based sensor, the MIP modifier has to form an homogenous, non-conductive and reproducible layer. Indeed, the imprinted film should allow the exchange of electrons between the electroactive probe and the electrode surface only through the target-mimetic cavities. The estimate thickness of the optimized MIP film on the gold disk electrodes was of about 200 nm [12] and its non-conductive nature was verified by cyclic voltammetry. However, the morphology and the properties of MIP do not depend only on the synthesis protocol (i.e., pre-complex mixture, electropolymerization conditions, etc.) but also on the electrode surface features (i.e., roughness, conductivity, etc.) [14,15].

In the present study, the possibility to apply a MIP synthesis protocol, developed on gold disk electrodes, to gold screen-printed electrodes (Au-SPE) was investigated, using a multi-analytical approach. Aiming to develop a portable and low-cost device, commercial Au-SPE readily available on the market were considered. Electrochemical techniques were employed to study the charge transfer on the modified electrode surface and to gather information about the imprinting process and overall performances of the sensor [16,17]. Imaging techniques were applied to map the morphology and properties of the bare electrode and the MIP-modified ones [15,18]. Thanks to the high concentration of fluorine in the template/target molecule, it was possible to visualize the MIP distribution by field-emission scanning electron microscopy–energy-dispersive X-ray spectrometry (FE-SEM–EDS) imaging. It is worth noting that this is the first report of direct visualization by elemental mapping of molecularly imprinted film on electrode surfaces.

The main goal of this study is to provide useful guidelines to easily redesign MIP sensors, stimulating the technological transfer of already existing bio(mimetic) sensors. The first part of the study was dedicated to the electrochemical study of the portable sensors and the evaluation of its performances. The second part was dedicated to the SEM/EDS study of the bare Au-SPE and the modified ones, investigating the impact of electrode surface features on the MIP morphology. A multi-analytical characterization protocol to be used in the optimization study was identified and considered as a fundamental step in the redesign process.

## 2. Materials and Methods

### 2.1. Materials, Electrochemical Apparatus and Characterization Techniques

Perfluorooctanesulfonic acid potassium salt (PFOS ≥ 98%), o-phenylenediamine (o-PD ≥ 98%), ferrocenecarboxylic acid (FcCOOH ≥ 97%) were purchased from Sigma-Aldrich (Overijse, Belgium). All other reagents were of analytical grade and solutions were prepared using MilliQ water.

Gold screen-printed electrodes 220AT (Au-SPE) were purchased from DropSens (Metrohm, Antwerp, Belgium). They are composed of a gold working electrode (3 mm diameter), a gold counter electrode and a silver pseudo reference electrode.

Cyclic voltammetry (CV), differential pulse voltammetry (DPV) and electrochemical impedance spectroscopy (EIS) were carried out using a Metrohm Autolab potentiostat/galvanostat (PGSTAT 302 N, Metrohm Autolab, Utrecht, The Netherlands) controlled by NOVA 1.1 software.

A field emission scanning electron microscope (FE-SEM) Zeiss Sigma|VP was operated with 20 kV accelerating voltage to collect electron micrographs of the samples; the instrument is equipped with an energy-dispersive X-ray spectrometer (EDS) Bruker Quantax 200, a system of the silicon drift detector (SDD) type with a 30 mm^2^ collection window. The element ratios were calculated with a standardless approach using the Bruker ESPRIT v.1.9 software, with the Interactive PB-ZAF algorithm.

Roughness parameters were calculated by data obtained with an AlphaStep 500 stylus-based surface profiler. Different scans, recorded by changing the scan speed, the sampling frequency, the scan length, the stylus force and the position on the sample surface, were analyzed. Scan length was always between 500 and 1000 µm.

Atomic force microscopy (AFM) images were obtained with an Asylum Research (Oxford Instruments Company, Abingdon, UK) Cypher instrument, used for both the topographical signal (collected in intermittent-contact mode) and the conductivity map collected with the conductive-AFM mode (C-AFM).

### 2.2. Preparation for Molecularly Imprinted Polymer (MIP) and Non-Imprinted Polymer (NIP) Sensors: From Synthesis to Template Removal

Au-SPE were washed with MilliQ water before use. The molecularly imprinted polymer (MIP) pre-complex solution was prepared by mixing 10 mM o-PD and 1 mM PFOS in 0.1 M acetate buffer pH 5.8 with 10% methanol (1:10 monomer:template ratio) and kept at 4 °C before use.

The MIP electropolymerization was performed in 100 µL drop of pre-complex solution by cyclic voltammetry, scanning 25 consecutive cycles in the potential window from 0.0 V to +1.0 V vs. pseudo Ag (Au-SPE internal reference) at a scan rate of 50 mVs^−1^. The non-imprinted polymer (NIP), used as a control experiment, was synthesized using the same protocol without adding the PFOS template in the pre-complex solution. All solutions were prepared fresh and stored at 4 °C for no more than 12 h.

After the electropolymerization, the modified electrodes were rinsed with 5 mL of MilliQ water, dried under air flow and stored at room temperature. The template was removed by letting 50 µL drops of methanol/MilliQ water (1:3, *v*/*v*) solution in contact with the working electrode for 5 min for three times reaching a total extraction time of 15 min. The electrode was rinsed with 1 mL of MilliQ water after the two first extraction steps and with 1 mL of methanol/MilliQ water (1:2, *v*/*v*) after the last one. The MIP/NIP electrodes were dried under air flow and kept at room temperature before use.

### 2.3. Electrochemical Characterization and Performance of MIP and NIP Sensors

The electrochemical characterization was carried out in 0.5 mM FcCOOH, 0.01 M ammonia buffer pH 8.4 by CV and EIS. The CVs were performed between −0.4 V and 0.6 V vs. pseudo Ag, 50 mVs^−1^ scan rate, 2 to 4 scans. The EIS measurements were performed between 0.1 MHz and 0.1 Hz, 0.01 V amplitude and bias potential determined by open circuit potential (OCP). Equivalent circuit fitting was performed with NOVA 1.1 software and all the data were verified with the Kronig-Kramers transformation for consistency [19,20].

The electrochemical measurements of PFOS were carried out in 100 µL drops. A series of 0.01 M ammonia buffer pH 8.4 solutions spiked with different concentration of PFOS (from 50 nM to 500 nM) were prepared. In this rebinding step, the 100 µL drops of PFOS solutions were left in contact with the modified electrodes for 15 min. After this incubation, the drops were washed away using 1 mL of methanol/MilliQ water (1:2, *v*/*v*) and the electrodes were tested in 100 µL of 0.5 mM FcCOOH, 0.01 M ammonia buffer pH 8.4 by performing CV and DPV. For the CV, the conditions mentioned above were applied, while for DPV the following parameters were used (previously optimized [12]) potential window between 0.0 V and 0.5 V vs. pseudo Ag, pulse width 25.0 ms, pulse amplitude 25.0 mV, increment potential 4.0 mV and scan rate 20 mVs^−1^.

The calibration plots were obtained by performing the rebinding step of four different solutions at increasing concentration of PFOS (50, 100, 250 and 500 nM). In between each step, the modified electrodes were accurately rinsed with 1 mL of methanol/MilliQ water (1:2, *v*/*v*). A baseline correction using the moving average (*n* = 1) tool within the NOVA 1.1 software was operated on the DPV voltammograms obtained. All the analyses were performed in triplicates.

Except where otherwise stated, all the potentials are referred to Ag pseudo reference (about −200 mV compared to SCE). All electrochemical experiments were performed at room temperature.

## 3. Results and Discussion

### 3.1. Electrochemical Characterization of MIP-Gold Screen-Printed Electrodes (Au-SPE)

The original protocol reported by Karimian et al. [12] was adapted to the commercial screen-printed gold electrodes (Au-SPE) aiming to fully exploit the potentialities of SPE. The electropolymerization was carried out in a drop of 100 μL to reduce the pre-complex solution volume. The percentage of methanol in the pre-complex solution and in the template removal step was drastically decreased because an excessive methanol content was found to affect the electrode performance, as showed in Supplementary Material Figure S1. After these first changes, the adapted protocol (fully described in Section 2.2) was utilized to obtain imprinted (MIP) and non-imprinted (NIP) polymers. An electrochemical characterization of the modified sensors was performed by CV and EIS.

For these preliminary tests, the electropolymerization patterns of both MIP and NIP polymers (compared in Supplementary Material Figure S2) were found to be consistent with the ones previously reported (see Figure 1 in [12]). The first electropolymerization CV cycle of the NIP showed two consecutive oxidation peaks at +0.3 V and +0.6 V as observed by Losito et al. [21]. For the MIP, the first cycle presented a unique peak at +0.3 V. This change in the electropolymerization pattern indirectly confirmed the complete dissolution of the PFOS in the pre-complex solution even in presence of a reduced methanol content.

**Figure 1:**
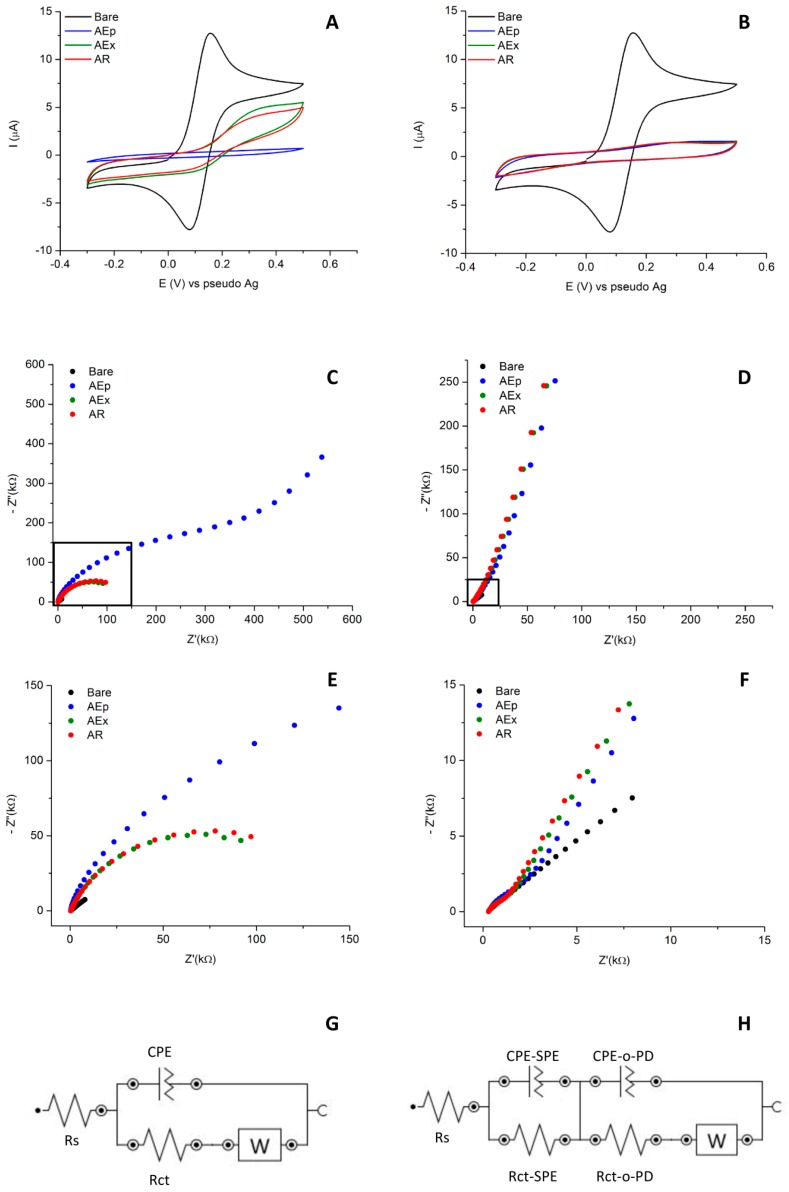
Electrochemical characterization study of imprinted (MIP) and non-imprinted (NIP) polymers at gold screen-printed electrode (Au-SPE). Cyclic voltammetry (CV) and Nyquist plots obtained after each step of the modified protocol for MIP ((**A**,**C**), respectively) and NIP ((**B**,**D**), respectively). The CVs were scanned at 50 mVs^−1^, while EIS spectra were recorded with an amplitude of 0.01 V and a bias potential determined by open circuit potential (OCP). All measurements were performed in 0.5 mM FcCOOH, 0.01 M ammonia buffer (pH 8.4). Zoom in of the Nysquit plot for MIP (**E**) and NIP (**F**) and equivalent circuits used to fit the EIS data (**G**,**H**). The abbreviations used in the legend indicate the following steps: *Bare*, unmodified Au-SPE (in black); *AEp*, after electropolymerization (in blue); *AEx*, after template extraction (in green); *AR*, after target rebinding (in red).

The step-by-step characterization reported in Figure 1 allowed to follow the formation of the imprinted film on the electrode surface. After the electropolymerization, both MIP and NIP modified electrodes showed a non-conductive behaviour as expected for the formation of a non-conductive polymer on their surfaces. The redox behaviour of the mediator is completely suppressed for both the MIP (blue curve in Figure 1A) and the NIP (blue curve in Figure 1B). However, the capacitive current of MIP and NIP electrodes had a different intensity: for the MIP it was about 6 ± 0.9 nA while for the NIP a higher value of about 17 ± 0.8 nA was recorded. This difference was simply ascribed to the different composition of the electropolymerization solution and in particular to the presence of the PFOS template in the MIP, which influences the properties of the final polymer.

After the template extraction, the redox activity of the mediator is again visible on MIP (green curve in Figure 1A) while it was still not present on NIP (green curve in Figure 1B, overlapped with red and blue curves). This behaviour is consistent with the fact that after extraction of the template the imprinted cavities are free again and thus allowing the electron exchange between the electrode surface and the mediator in solution. However, the electron transfer (ET) is heavily influenced by the presence of the MIP: the ΔEp increases (from 65 mV to 145 mV) with a concurrent decrease of peak intensity for both anodic and cathodic processes. As expected, for the NIP there is no difference before and after extraction since there are no empty imprinted cavities.

After the rebinding of 500 nM PFOS, a decrease in the FcCOOH signal was recorded at MIP-Au-SPE (red curve in Figure 1A). This can be considered as an indication of the successful recognition event between the target molecule and the imprinted cavities, as previously stated [12]. Indeed, once the cavities are re-loaded with the target the interaction of the redox mediator with the electrode surface and consequently its signal will decrease again. As expected, no changes were observed at NIP-Au-SPE (red curve in Figure 1B). This seems to confirm the MIP recognition capabilities towards PFOS and suggests the success of the imprinting protocol.

Looking at the EIS results for both MIP and NIP, it is possible to acquire additional information about the polymer formation and characteristics. The Nyquist plots for both MIP (Figure 1C) and NIP (Figure 1D) showed the successful deposition of the polymer film. The spectra of the bare Au-SPE electrodes (black dots in Figure 1E,F) can be fitted to a Randles equivalent circuit (Figure 1G) where R_s_ is the uncompensated solution resistance, CPE the constant phase element used to model the double layer capacitance, R_ct_ the charge transfer resistance and W the Warburg impedance, that account for the semi-infinite diffusion of the electroactive species from the bulk of the solution towards the surface of the electrode.

After the electropolymerization, the impedance behaviour of the MIP changes: the blue curve in Figure 1C shows a very depressed semicircle at higher frequencies and a linear trend at lower frequencies. The radius of the semicircle is linked to the charge transfer resistance: since there is an insulating modifier on the surface of the electrode, the ET between the mediator and the electrode is heavily impaired.

After the extraction (green curve in Figure 1C), the semicircle radius decreases again, as now the cavities are free and the mediator can reach again the electrode surface. After rebinding of 500 nM of PFOS the Nyquist plot for the MIP (red curve in Figure 1C,E) does not show perceptible changes: indeed, an increase in the R_ct_ was expected since the cavities are again occupied.

However, the PFOS concentration is very low compared to the original pre-complex solution (1 mM) and does not affect the impedance behaviour of the sensor. For the NIP, instead, the Nyquist plot after electropolymerization (blue curve in Figure 1D) shows a modest increase in the semi-circular part at higher frequencies (see also blue curve in Figure 1F) while the linear part is very steep and starts at relatively high frequencies compared to the MIP. Again, no sensible changes are present after extraction and rebinding of 500 nM of PFOS as already shown by CV (see Figure 1B). The impedance data for both the MIP and NIP sensors could be fitted by the equivalent circuit reported in Figure 1H. The circuit account for the presence of a non-conductive polymeric layer on top of a conductive electrode surface [22,23]. CPE-SPE and R_ct_-SPE are, respectively, the constant phase element and the charge transfer resistance of the Au-SPE, while CPE-o-PD and R_ct_–o-PD refer to the o-PD polymer film. The values of the charge transfer resistance (summarized in Supplementary Material Table S1) for both MIP and NIP confirm this interpretation. While the R_ct_ –o-PD of the NIP remain constant for each step (c.a. 2 kΩ), the MIP present very high values after electropolymerization (125 kΩ), that diminish sensibly after extraction (around 20 kΩ). This first characterization of the MIP-Au-SPE gave positive results suggesting the feasibility of the optimization of the original protocol [12] on screen-printed electrodes. To validate the proposed protocol, the analytical performance of the imprinted sensors was tested and compared to the original sensor.

### 3.2. Performance of MIP-Au-SPE

**Figure 2:**
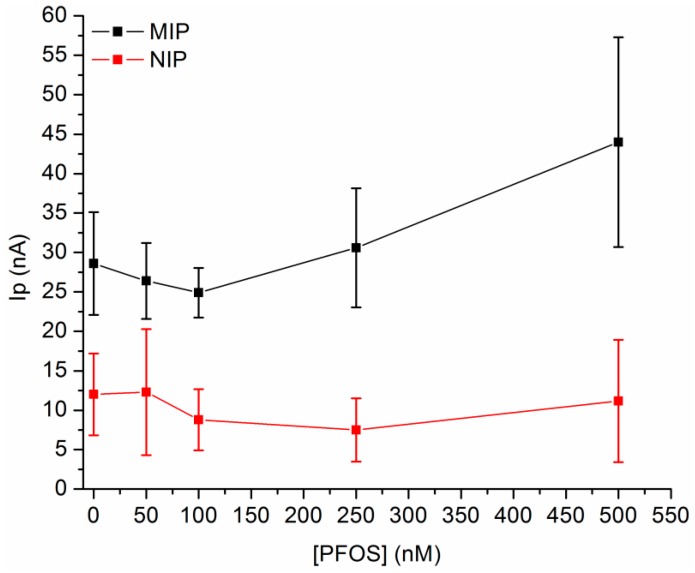
Calibration plots of imprinted (MIP, black) and non-imprinted (NIP, red) polymers at gold screen-printed electrodes obtained after the rebinding of 0, 50, 100, 250 and 500 nM of PFOS. The signal were recorded by DPV in the potential window between 0.0 V and 0.5 V vs. pseudo Ag, with a pulse width of 25.0 ms, a pulse amplitude of 25.0 mV, an increment potential of 4.0 mV and a scan rate of 20 mVs^−1^ in 0.5 mM FcCOOH, 0.01 M ammonia buffer (pH 8.4). The values presented are the average of three measurements and the error associated to the standard deviation.

The performance of the imprinted sensors were evaluated by differential pulse voltammetry (DPV), NIP-Au-SPE was used as control experiment. Four different concentrations of PFOS (50, 100, 250 and 500 nM) within the linear range previously reported [12] (from 0.1 nM to 1.5 µM) were selected. The calibration plot, obtained from triplicate measurements, at MIP-Au-SPE showed a poor reproducibility and an unexpected trend (see Figure 2). Instead of a linear decrease in signal at increasing PFOS concentrations, a non-linear evolution with a net signal increase at 500 nM was observed. For 50 and 100 nM, signals with current intensities slightly lower than the unloaded MIP were recorded and these variations were not considered relevant because of the high error associated. For 250 and 500 nM, the increased signals and associated errors might suggest the instability of the obtained modification and the partial removal of the polymer from the electrode surface. The relatively high errors associated with the measurements depend mainly on the highly variable responses of the different MIP-Au-SPE sensors and can possibly be ascribed to the variability in the electrode surface properties. As a control experiment, NIP-Au-SPE were also tested showing no relevant variations in the signal intensity after the different rebinding, as expected. The high error associated to the values presented can be explained with the lack of target-template cavities and the occurrence of adsorption phenomena [12]. No net signal increases were observed for 250 and 500 nM and this was ascribed to the higher stability of the non-imprinted polymer itself. Also, the PFOS affinity for the bare Au-SPE was tested, operating the rebinding on unmodified electrodes. From the calibration plot obtained (in Supplementary Material Figure S3), minimal signal variations (with a Δi for 50 and 500 nM PFOS solutions of about 2.5 ± 0.5 µA) were recorded with an inversely proportional trend between the target concentration and the mediator signal. The higher reproducibility of the measurements at bare Au-SPE in comparison to MIP/NIP-Au-SPE indirectly suggest the low reproducibility and instability of polymeric modifiers, while the trend registered confirm the PFOS affinity for the Au-SPE surface and the importance of considering this parameter in further optimizations.

The analysis of the MIP-Au-SPE performances allowed to define the shortcomings of the used protocol and to underline the need of further optimization. Trying to understand the reasons behind these poor performances, the morphological characterization of the bare electrode surface and the MIP and NIP modified sensors was performed.

### 3.3. Morphological Characterization

#### 3.3.1. Surface Analysis of Unmodified Au-SPE

**Figure 3:**
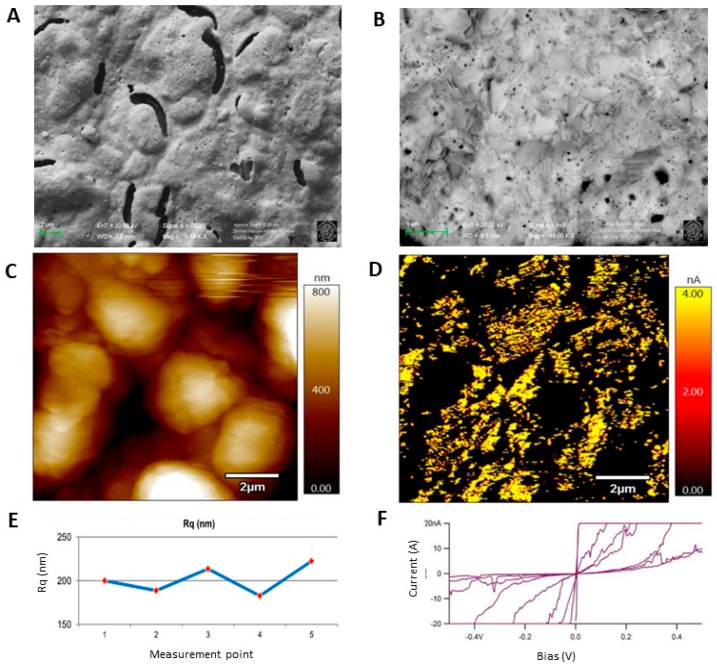
Surface characterization of unmodified gold screen-printed electrode imaged with: field-emission scanning electron microscopy (FE-SEM) at 9.16 kx using secondary electrons (**A**) and 40 kx using backscattered electrons (**B**), both at 20.00 kV; atomic force microscopy (AFM) topography (**C**) and conductivity (**D**) modes. AFM correlated graphs showing the roughness (**E**) and the conductivity (**F**) measured in different points.

The roughness and the electronic properties of the bare Au-SPE were investigated by FE-SEM, profilometry and AFM. From FE-SEM micrographs, it was possible to observe a heterogeneous surface, characterized by swells and holes as can be seen in the secondary electron micrograph reported in Figure 3A. The real geometry of this complex surface was further characterized estimating the surface roughness in the micrometer and nanometer range. By means of the stylus surface profiler, a root mean square roughness, Rq, of (0.75 ± 0.10) µm was calculated from the obtained data in Supplementary Material Figure S4, as defined in [24]; the uncertainty is related to a 95% confidence interval. The estimated Rq (<1 µm) suggested the importance to characterize the surface roughness is in the nanometer range. This latter might be related to the disordered growth of Au crystallites and it was investigated by AFM topographical images. Figure 3C showed that the total height of the swells is about 0.8 µm. AFM was used also to calculate the Rq, on five different sites imaged (one is reported in Figure 3C). A mean value of 200 nm was obtained. The variation of the R_q_ value from site to site reported in Figure 3E was about 15 nm. This R_q_ value, (200 ± 15) nm, suggested the presence of a highly variable surface. Moreover, at higher magnification, a platelet-shaped fine structure was imaged with FE-SEM, as shown in Figure 3B. It was noticed that even though the platelets have different orientation in different sample areas, in a single area they tend to be aligned with each other. The elemental composition of the bare electrode surface was also investigated by EDS analysis (Supplementary Material Figure S5) to verify the possible presence of other metals in amalgam with Au that could influence the final properties of the electrode [25]. However apart from gold, no other elements were found.

From AFM measurements, it was possible to map the heterogeneous conductivity of these electrodes. It was observed that areas with smaller conductivity correspond to the swells in topographical image, while the holes present a relatively higher but non-uniform conductivity, as can be seen by comparing Figure 3C,D (acquired during the very same scanning through different channels of the instrument). This behaviour leads to different current-voltage profiles in the different points of the surface, as shown by the curves in Figure 3F. The profiles reported have different slopes and ohmic intervals ranging from (12 ± 5) mV to (670 ± 130) mV.

The surface characteristics of the Au-SPE (220AT, Dropsens) differs deeply from the homogenous, approximately flat bulk gold disk electrode surface. The heterogeneous conductivity of the surface might influence the polymer growth.

#### 3.3.2. Surface Analysis of MIP and NIP

**Figure 4:**
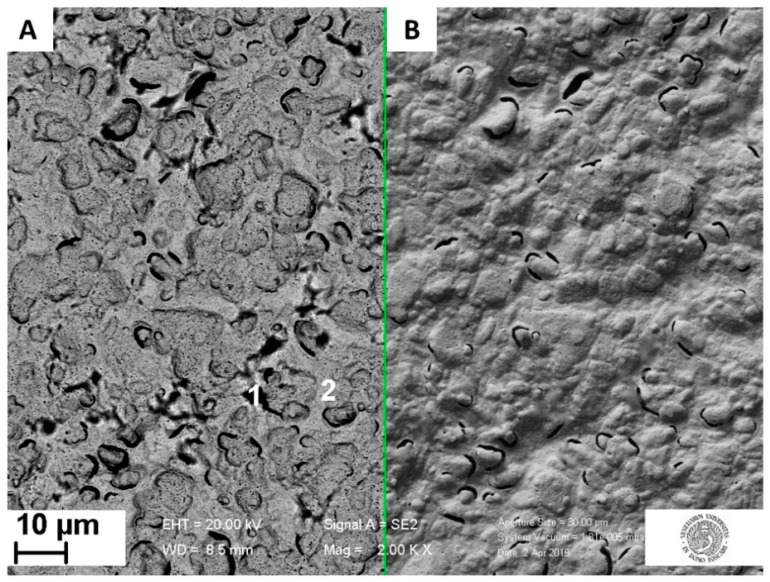
Surface of a MIP modified Au-SPE imaged with FE-SEM using the backscattered electrons (**A**) and secondary electrons (**B**) at 20.0 kV with a magnification of 2 kx; inset: numbers indicating the points where energy-dispersive X-ray spectrometry (EDS) spectra (reported in Supplementary Material Figure S5) were recorded.

The MIP distribution after polymerization was investigated with backscattered and secondary electrons. By comparing the images of the same area obtained with these two signals it was possible to recognize the polymer distribution. Due to the dependence of backscattered electron (BSE) signal to the average atomic number Z of the sample material hit by beam (primary) electrons [9,10], in BSE micrographs the polymer is darker than higher-Z areas, like bare gold ones. On the other hand, the secondary electron (SE) signal is much less sensitive to the average atomic number [26,27]. Therefore, in BSE images it was possible to recognize areas richer in polymer with respect to the surroundings as dark areas. However, each dark area cannot be identified as a polymer, because also holes on the bare electrode surface appear dark; nonetheless they also appear dark in SE images, and this allowed to discriminate which dark areas in BSE micrographs were holes and which were not. With this approach, the comparison of BSE (Figure 4A) and SE (Figure 4B) of the same area of the sample highlighted the presence of low-Z areas, which reasonably were polymer areas (due to the nature of the sample).

The EDS reported in Supplementary Material Figure S6 confirmed this hypothesis. Indeed, these dark areas are rich in C and F atoms, which are present in the MIP, while the surrounding light areas give a lower C peak and almost no F peak at all. Thus, this SEM/EDS analysis showed that the polymer is mainly growing in islands on the surface of the working electrode.

**Figure 5:**
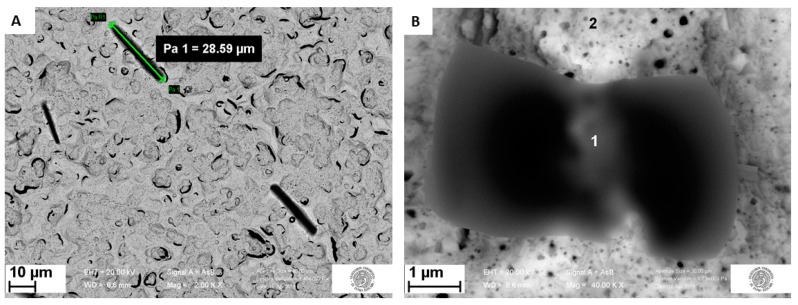
Examples of MIP heterogenous distribution after electropolymerization with the presence of polymer ‘islands-like’ aggregates. Overall distribution at 2.00 kx, with green arrow and label indicating the island length of about 28.6 µm (**A**). Zoom on a polymeric island at 40.00 kx, where the numbers indicate the EDS spectra acquisition points (EDS spectra reported in Supplementary Material Figure S6) (**B**). Both images were obtained using backscattered electrons.

Other examples of these islands are visible in Figure 5A, which shows that in some cases the polymer grows in rod shape, with a length up to almost 30 µm. The relatively regular shape of these islands might be ascribed to the crystalline nature of the polymer in these aggregates (OPD crystals have an orthorombic lattice [28]).

The presence of polymer islands on the sample surface does not imply that the polymer is present only in those areas: both EDS spectra (Supplementary Material Figure S6) of point 2 from Figure 4 and Figure 5 show clearly that a lower peak of carbon is present also outside the islands, suggesting the polymer presence on the rest of the surface.

In Figure 5B, a close-up image of a single island is reported, associated with the EDS spectra in Supplementary Material Figure S7. The presence of fluorine in the EDS spectra from the islands (Supplementary Material Figures S6(A-1) and S7(B-1)) confirm the successful entrapment of the PFOS in the polymer matrix with a weight concentration ratio between F and C, $c_{F}/c_{C}$, of 0.94. In the spectra (Supplementary Material Figures S6(A-2) and S7(B-2)) of the region outside the polymer islands a weak fluorine peak was observed, revealing the presence of the PFOS also out of the islands; with a $c_{F}/c_{C}$ of 0.07. These observations suggested that the pre-complex electropolymerization is not homogenous and that the PFOS is preferentially associated within polymeric islands.

**Figure 6:**
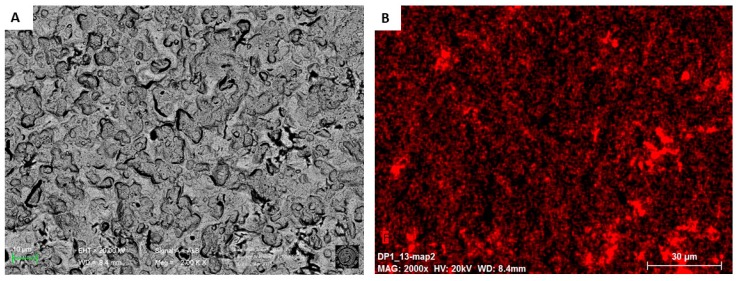
Examples of MIP heterogenous distribution after electropolymerization: image obtained with backscattered electron (BSE), 20.00 kV and a magnification of 2.00 kx (**A**) and the corresponding fluorine map (**B**), whose most intense areas are at the same locations as low Z island in BSE micrograph. This map was elaborated applying a smooth filter with kernel size 3 to the data collected by the detector.

Thanks to the high content of fluorine, it was possible to map the template distribution on the electrode surface by means of an X-ray map of the fluorine peak. In Figure 6, a backscattered micrograph is compared with the fluorine map of the same area. The latter clearly highlights the places where the fluorine is more concentrated, which not surprisingly mainly corresponds with the dark islands in the BSE micrograph. Moreover, in the X-ray map a more or less continous background can be seen, thus confirming the presence of inhomogeneous MIP distribution also outside the islands.

The same set of measurements was performed also on the NIP-Au-SPE. No traces of fluorine were observed and a similar heterogeneous polymer distribution was suggested by the presence, in the carbon X-ray map, of higher concentration areas similar to those found in the fluorine map in Figure 6. These maps were obtained with an acquisition time optimized to 6 min, a reasonably short time for the 30 mm^2^ window SDD detector used. The optimization of the instrument settings helped increasing the collection efficiency, reducing the measuring time needed to reach the requested signal-to-noise ratio in the map.

## 4. Conclusions

Sensor redesign is often a limiting step towards the technological transfer of electrochemical sensors for in situ environmental monitoring. In this work, the impact of electrode surface features in the redesign of an MIP-sensor for PFOS have been clarified. The possibility to adapt the MIP modification protocol to portable electrodes was first investigated using CV and EIS to characterize the MIP synthesis steps. The results obtained were encouraging even though the final sensor performance was not comparable to the MIP on bulk electrodes.

The extended SEM–EDS study performed to describe the morphology and distribution of the MIP modifiers allowed to visualize the formation of polymer island with an higher content of template. These information helped to interpret the data obtained from the first electrochemical study of the sensor. The poor reproducibility at Au-SPE can be ascribed to the heterogenous distribution of the MIP polymer due to the roughness of this substrate. Indeed, commercial gold screen-printed electrodes were found to have a highly rough surface which did not allow a homogenous polymerization of MIP. This could be one of the main reason behind the poor reproducibility of MIP-Au-SPE responses. These results suggested the need to continue this redesign process by focusing on the electrode surface, including a surface activation treatment to improve the conductivity or to test other Au-SPEs produced with different fabrication tecniques (i.e., sputtered).

The unique possibility to perform a complete morphological study, visualizing the MIP distribution at the electrode surface, should be further applied in the next optimization steps of the redesign and extended to the study of other MIP with templates rich in fluorine (particularly the per- and poly fluoroalkyl substances). The re-optimization study presented here showcased several of the possible issues in the technological transfer of imprinted electrode modifiers, stressing once more the need for a deeper connection between fundamental research done in the laboratory and envisioned commercial applications.

## Supplementary Materials

The following are available online at https://www.mdpi.com/1424-8220/19/20/4433/s1: Figure S1: Effect of methanol content on the performances of Au-SPE. Figure S2: Comparison of the electropolymerization pattern of imprinted (A) and non-imprinted (B) polymers on Au-SPE. Table S1: Charge transfer resistances (R_ct_-o-PD) for the MIP and NIP: AEp after electropolymerization, AEx, after extraction, AR, after rebinding. Figure S3: Calibration plot of PFOS at bare Au-SPE. Figure S4: Example of surface scan recorded by the stylus profiler. Figure S5: SEM image of the bare working electrode surface of an Au-SPE and EDS spectra of the point 1 and 2 in the image. These analysis are representative of the whole working electrode surface. Figure S6: EDS spectra of the point 1 (A-1) and 2 (A-2) in Figure 4A. Figure S7: EDS spectra of the point 1 (B-1) and 2 (B-2) in Figure 5B.
