# Supplementary material for: Redesigning an Electrochemical MIP Sensor for PFOS: Practicalities and Pitfalls
Source: Sensors (Basel). 2019 Oct 13;19(20):4433. doi: 10.3390/s19204433 (PMC6832149; doi:10.3390/s19204433)
Supplement: Supplementary file 1 [file sensors-19-04433-s001.pdf]

sensors

Supplementary Materials:

Redesigning an Electrochemical MIP Sensor for PFOS: Practicalities and Pitfalls

Giulia Moro [1,2], Davide Cristofori [1,3], Fabio Bottari [2], Elti Cattaruzza [1], Karolien De Wael [2] and Ligia Maria Moretto [1,*]

[1] Department of Molecular Sciences and Nanosystems, Ca' Foscari University of Venice, Via Torino 155, 30172 Mestre, Italy; giulia.moro@unive.it (G.M.); dcristofori@unive.it (D.C.); cattaruz@unive.it (E.C.)

[2] AXES Research Group, Department of Chemistry, University of Antwerp, Groenenborgerlaan 171, 2020 Antwerp, Belgium; fabio.bottari@uantwerpen.be (F.B.); karolien.dewael@uantwerpen.be (K.D.W.)

[3] Centre for Electron Microscopy "Giovanni Stevanato", Università Ca' Foscari di Venezia, Via Torino 155, 30172 Mestre, Italy

* Correspondence: moretto@unive.it; Tel.: +39-041-234-8585

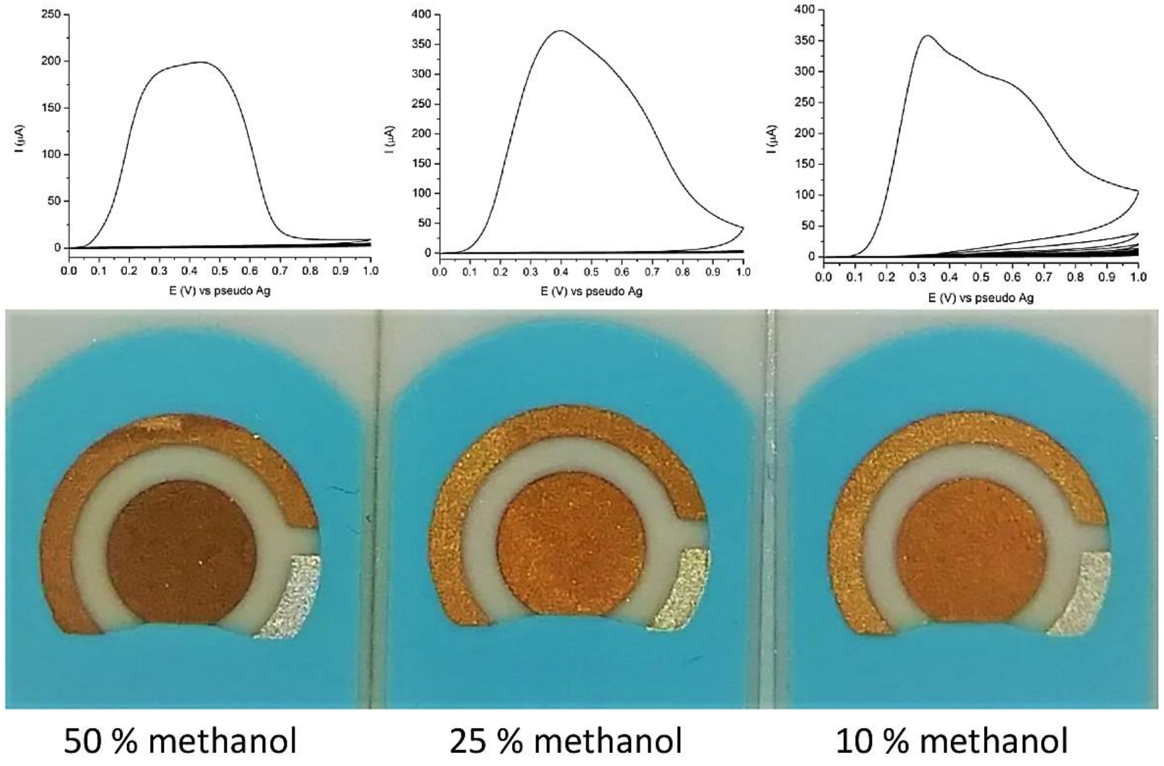

Figure S1. Effect of methanol content on the performances of Au-SPE.

With 50% methanol the first CV cycle of the electropolymerization presents a broad oxidation peak with a relative low current intensity (about 200 μA) and the working electrode (WE) appears darker at the end of the process. With 25% methanol the WE is slightly darker and the electropolymerization pattern presents a more defined oxidation peak. Only reducing the methanol concentration to 10%, the expected electropolymerization pattern with a first CV cycle characterized by multiple oxidation peaks was recorded. This was also the minimal concentration applicable to assure the complete dissolution of the PFOS, the target-template.

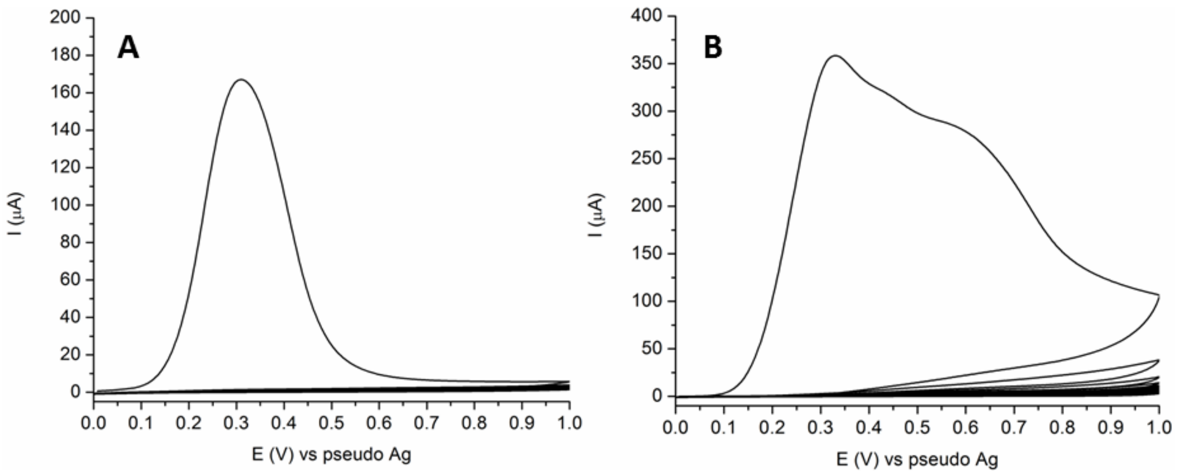

Figure S2. Comparison of the electropolymerization pattern of imprinted (**A**) and non-imprinted (**B**) polymers on Au-SPE.

Table S1. Charge transfer resistances (R_{ct}-o-PD) for the MIP and NIP: AEp after electropolymerization, AEx, after extraction, AR, after rebinding.

**MIP**	**R_{ct}-o-PD (kΩ)**	**χ^2**
AEp	125	0.03
AEx	21.7	0.02
AR	20.5	0.005
**NIP**	**R_{ct}-o-PD (kΩ)**	**χ^2**
AEp	2.37	0.005
AEx	2.15	0.03
AR	2.32	0.005

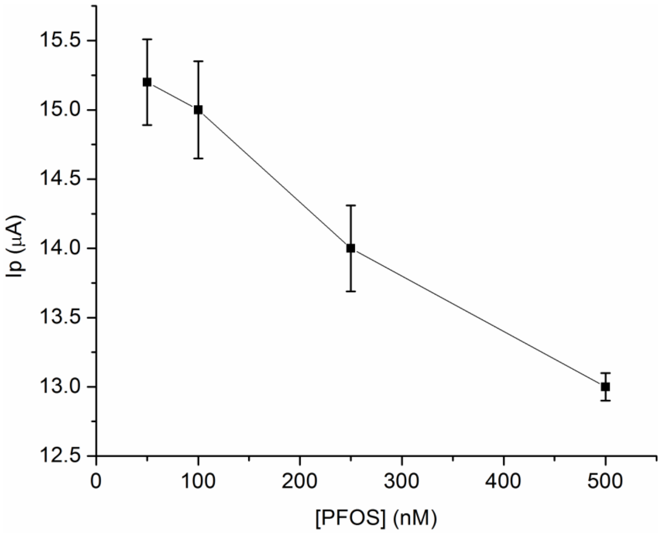

Figure S3. Calibration plot of PFOS at bare Au-SPE.

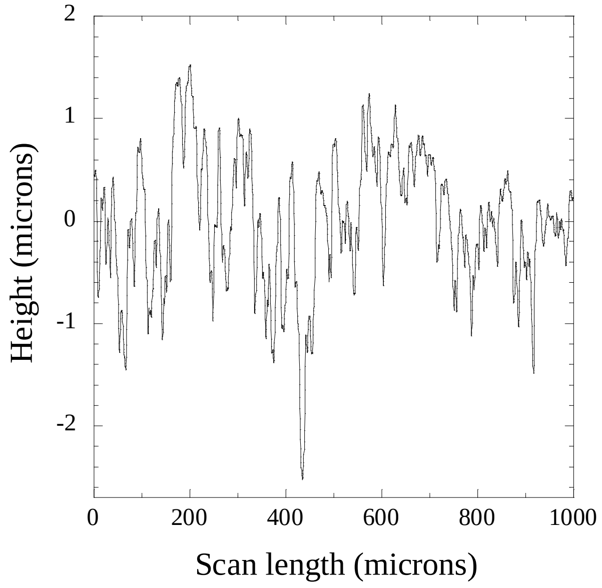

Figure S4. Example of surface scan recorded by the stylus profiler.

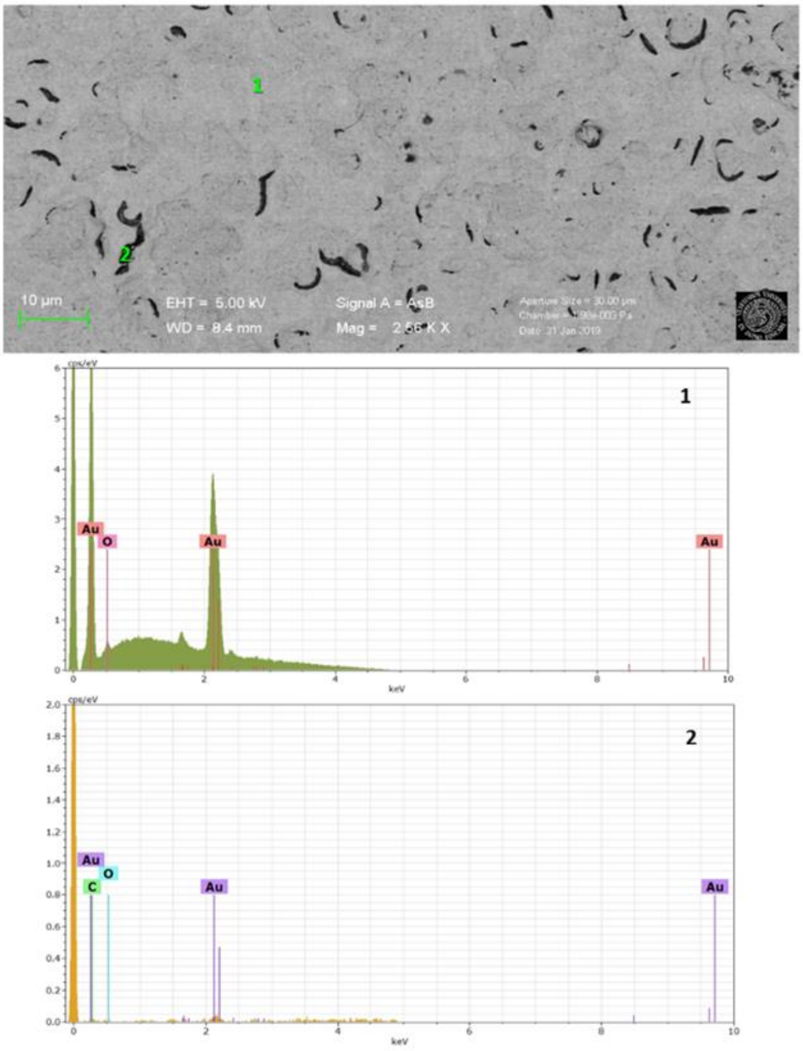

Figure S5. SEM image of the bare working electrode surface of an Au-SPE and EDS spectra of the point 1 and 2 in the image. These analysis are representative of the whole working electrode surface.

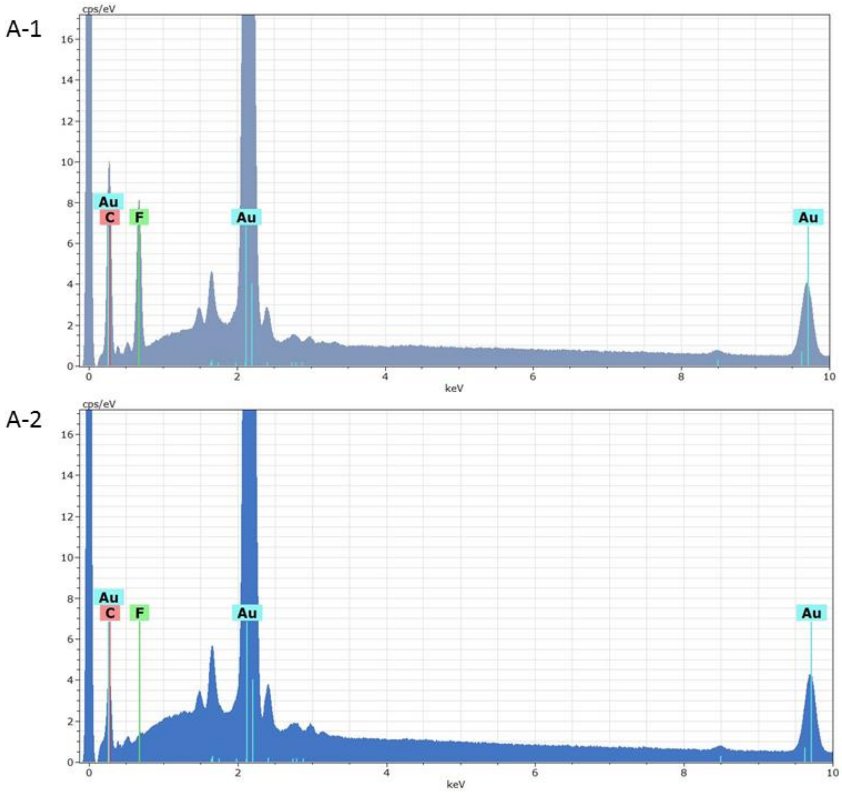

Figure S6. EDS spectra of the point 1 (A-1) and 2 (A-2) in Figure 4A.

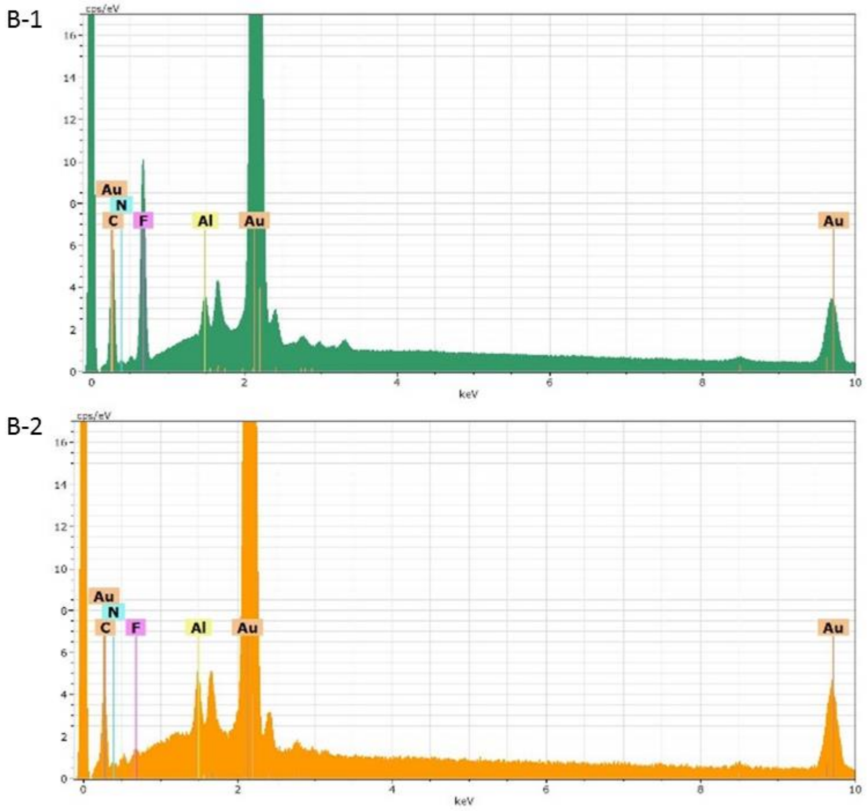

Figure S7. EDS spectra of the point 1 (B-1) and 2 (B-2) in Figure 5B.
